# Study of Visual Function in Adult Epileptic Patients on Sodium Valproate or Carbamazepine Monotherapy

**DOI:** 10.7759/cureus.4553

**Published:** 2019-04-27

**Authors:** Tong Jong Haw Matthew, John Tharakan, Evelyn Tai, Adil Hussein

**Affiliations:** 1 Ophthalmology, School of Medical Sciences, Universiti Sains Malaysia, Kubang Kerian, MYS; 2 Neurosciences, School of Medical Sciences, Universiti Sains Malaysia, Kubang Kerian, MYS

**Keywords:** carbamazepine, sodium valproate, color vision, visual field, contrast sensitivity, retinal fiber layer thickness

## Abstract

Objective

Epilepsy is a debilitating disease. Visual function changes have been reported and may be attributed to the epileptic changes or as a result of medication side effect. Sodium valproate and carbamazepine are both first line anti-epileptic medications used in Malaysian health care. Sodium valproate inhibits glutamate and γ-aminobutyric acid (GABA) transaminase while carbamazepine acts on the sodium channel - both are an important part of the retina. This study aimed to compare the visual functions of epilepsy patients on carbamazepine or sodium valproate monotherapy.

Design

A cross-sectional study was conducted at a tertiary hospital between June 2016 and November 2018.

Methods

Patients with idiopathic epilepsy that fulfill the inclusion and exclusion criteria were recruited from the neurology clinic. They were divided into two groups and underwent complete eye examinations. Visual functions such as color vision testing, contrast sensitivity, visual field and retinal nerve fiber layer measurement were subsequently performed. Statistical analysis was done using Statistical Package for the Social Science, version 24 (SPSS Inc, Chicago, IL, USA).

Results

A total of 100 patients (sodium valproate: 50 patients; carbamazepine: 50 patients) were recruited for the study. There were no statistically significant changes in anatomical or visual function between the sodium valproate and carbamazepine group. However, patients from both groups displayed color vision defect in the blue and green axes. Changes in color vision could indicate early retina toxicity secondary to the medication. Although there were no visual field changes, patients recorded a slight reduction of mean deviation. Changes of mean deviation could be attributed to the side effect of medication or the disease process.

Conclusions

Epileptic patients taking sodium valproate or carbamazepine did not demonstrate statistically significant change in visual function.

## Introduction

Epilepsy is a debilitating disease with a considerable economic burden. The continent of Asia is home to some of the world’s largest and poorest population with estimates of 25 million epileptics [1]. A recent study placed the prevalence of epilepsy in Malaysians at an estimate of 12.3% to 25.3% [2].

Epilepsy occurs due to a spontaneous, uncontrolled electrical discharge in the brain. The postulated pathway for the development of epilepsy includes imbalance in the neurotransmission signaling pathway and ion channel receptors. Glutamate and γ-aminobutyric acid (GABA) are important neurotransmitters in the brain. It is hypothesized that imbalance between the glutamate-mediated excitation and GABA-mediated inhibition results in the occurrence of seizure [3-6]. Similarly, ion channels are proteins on lipid membrane which allow selective movement of ions across cells [7,8]. Cation channels generate action potential which contribute to axon excitability, while anions are inhibitory. Imbalance between ion channels is also postulated to cause epilepsy [9].

Treatment of epilepsy generally involves antiepileptic drugs (AEDs), of which sodium valproate and carbamazepine are the first-line treatment of epilepsy in Malaysia. Sodium valproate functions by acting on GABA transaminase while carbamazepine acts by inhibition of voltage-dependent sodium channels [8,10]. Visual function-related complications, which have been reported in epileptic patients taking sodium valproate, include an increase of total error scoring (TES) when testing color vision with the Farnsworth Munson D-100. For epileptic patients taking carbamazepine, visual disturbances which have been reported include color vision, change in contrast sensitivity and change in visual field.

This study aimed to compare visual functions of epilepsy patients on carbamazepine or sodium valproate for early identification of retina toxicity.

## Materials and methods

A cross-sectional study was conducted in Hospital Universiti Sains Malaysia (HUSM), Kelantan between June and November 2018. The study was a collaboration between the ophthalmology department and the neuromedical department. The study followed the guidelines set by the Declaration of Helsinki and was approved by the ethical committee (USM/JEPeM/16090275). A total of 100 patients were recruited for the study. Eligible patients had idiopathic epilepsy, were at least 18 years old and legally able to give consent for participation. They also needed to be on either sodium valproate or carbamazepine monotherapy treatment for at least six months. Compliance to medication was measured via therapeutic drug monitoring (TDM) done within one year.

Exclusion criteria included best corrected visual acuity worse than 6/9, pre-existing visual defect, congenital color vision defect (screening via Ishihara pseudoisochromatic plates), and underlying ocular disease such as glaucoma and diabetic retinopathy. Patients with history of eye trauma, ocular or orbital surgery and previously treated with Vigabatrin were also excluded. Patients with any form of birth trauma or related complications, organic causes of neurological disorders, medical conditions, for example, hypertension, diabetic mellitus, psychiatric disorders, metabolic disorders, vitamin B12 deficiencies and diseases which could cause disturbance of visual function were also excluded.

All participants meeting the study criteria underwent comprehensive eye examination after written consent was obtained to exclude any eye pathologies. They were subsequently divided into two groups - those taking sodium valproate and those taking carbamazepine. Testing was performed monocularly; color vision was done using the Farnsworth Munson D-100, contrast sensitivity using the Pelli-Robson chart, and visual field using the automated Humphrey visual field (HVF) Swedish Interactive Thresholding Algorithm (SITA) with the parameter set at central 30-2. Measurement of the retinal nerve fiber layer (RNFL) was done with the spectral-domain optical coherence tomography (Cirrus HD-OCT; Carl Zeiss Meditec, Dublin, CA, USA).

Statistical analysis was carried out using Statistical Package for Social Science (SPSS Inc, Chicago, IL, USA) version 24. All values were tested for normal distribution and equal variances. Numerical variables were presented using mean and standard deviation if the data were normally skewed; while the median and interquartile range were used to present skewed numerical data. Categorical variables were presented using frequency and percentage. Pearson Chi-square and Fisher Exact test were used to test the association between two drug groups and the independent variable. Level of significance is set at p < 0.05. All tests were two-sided.

## Results

A total of 100 epileptic patients (50 patients for sodium valproate and 50 patients for carbamazepine) were recruited. Subjects had a mean age of 32.22 years (SD: 11.78) and a median age of 30 years. More than half of them were male (60.0%). Patients had a mean and median duration of medication of 12.07 years and nine years, respectively. The distribution of duration of medication was slightly skewed to the right. The present study compared the mean value and proportion of each clinical and demographic factor between carbamazepine and sodium valproate groups and found no significant difference between both groups (p > 0.05). Table 1 shows characteristics of study subjects.

**Table 1:** Demographic data of the study population (n = 100). ^a ^Independent t-test; ^b ^Pearson Chi Square; ^c ^Fisher Exact test SD: Standard deviation ^1 ^Data were positively skewed.

	All	Carbamazepine	Sodium Valproate	p-value
	(n = 100)	(n = 50)	(n = 50)	
Age^1 ^(year)				
Mean (SD)	32.22 (11.78)	31.50 (12.02)	32.94 (11.61)	0.544^a^
Median	30.00	29.50	31.50	
Range	18.00–67.00	18.00–60.00	18.00–67.00	
Gender (n, %)				
Male	60 (60.0)	29 (58.0)	31 (62.0)	0.683^b^
Female	40 (40.0)	21 (42.0)	19 (38.0)	
Race (n, %)				
Malay	95 (95.0)	48 (96.0)	47 (94.0)	>0.950^c^
Chinese	4 (4.0)	2 (4.0)	2 (4.0)	
India	1 (1.0)	0	1 (2.0)	
Duration of medication^1 ^(year)				
Mean (SD)	12.07 (9.71)	11.34 (9.94)	12.80 (9.51)	0.455^a^
Median	9.00	8.00	10.00	
Range	2.00–46.00	2.00–45.00	2.00–46.00	

The results of color vision, visual field, and contrast sensitivity testing, as well as retinal nerve fiber layer thickness are summarized in Table 2.

**Table 2:** Comparison of visual function between carbamazepine and sodium valproate (N = 100). HVF: Humphrey visual field; RE: Right eye; LE: Left eye; RNFL: Retinal nerve fibre layer; MD: Mean deviation; SD: Standard deviation; S: Superior; I: Inferior; N: Nasal; T: Temporal. ^1 ^Independent t-test was applied, normality and equal variance assumptions were checked. ^2 ^Fisher Exact test. ^3 ^Equal variances were not assumed. ^4 ^Data presented as frequency (percentage).

	All (n = 100)	Carbamazepine (n = 50)	Sodium Valproate (n = 50)	p-value^1^
	Mean (SD)	Mean (SD)	Mean (SD)	
Colour Vision^4^				
Normal	88 (88.0)	44 (88.0)	44 (88.0)	>.950^2^
Blue axes	11 (11.0)	6 (12.0)	5 (10.0)	
Green axes	1 (1.0)	0	1 (2.0)	
Contrast Sensitivity^4^				
Normal	100	50	50	NA
Abnormal	0	0	0	
HVF (MD, SD)				
HVF RE	-3.37 (6.79)	-4.28 (8.62)	-2.46(4.12)	0.180
HVF LE	-2.48 (3.20)	-2.80 (-3.49)	-2.15(2.87)	0.312
RNFL Thickness (μm, SD)				
RE RNFL S	119.38 (11.02)	120.52 (9.39)	118.24 (12.43)	0.303
RE RNFL N	69.90 (7.48)	70.54 (8.67)	69.26 (6.08)	0.395
RE RNFL I	118.95 (12.98)	119.10 (13.24)	118.80 (12.85)	0.909
RE RNFL T	64.08 (10.14)	63.84 (11.40)	64.32 (8.80)	0.814
LE RNFL S	121.42 (10.70)	120.52 (11.35)	122.32 (10.04)	0.403
LE RNFL N	68.52 (8.64)	68.14 (9.49)	68.90 (7.78)	0.662
LE RNFL I	119.95 (13.85)	121.90 (17.33)	118.00 (8.91)	0.161^3^
LE RNFL T	63.69 (7.89)	63.92 (7.63)	63.46 (8.22)	0.772

Color vision testing revealed that 12% of patients in the carbamazepine group developed errors along the blue-yellow axes, while 10% of subjects in the other group had a similar problem. None of the carbamazepine group had errors along the green axes, while one patient from sodium valproate group had this error. Overall, there was no significant difference in color vision abnormalities between the two groups. Likewise, there was no statistically significant difference in visual field and retinal nerve fiber layer between both groups. In term of contrast sensitivity, none of the subjects was found to be abnormal.

## Discussion

The results of this study can be divided into color vision, visual field, contrast sensitivity, and RNFL. Patients from both groups also denied any change in color vision after commencing medication. However, patients from both groups demonstrated similar color vision defects on pseudo-isochromatic testing. This finding reflects the results of other studies, in which a similar color vision defect was demonstrated among patients on similar AEDs [11-18]. Sorri et al. noticed a statistically significant color vision change along the blue axes for patients on sodium valproate when compared to controls. However, there were no correlations between color vision changes and duration, cumulative dose or plasma concentrations of sodium valproate [18].

In contrast, Bayer et al. demonstrated that epileptic patients taking sodium valproate had normal color vision [11]. In the same study by Bayer et al., epileptic patients taking carbamazepine were found to display errors along the blue-yellow axes [11]. However, Bayer et al. failed to demonstrate significant correlations between serum concentration of carbamazepine and color vision [11]. Changes in color vision can indicate alteration of normal physiological retina function or be a sign of retina toxicity [19]. The action of AED on altering neurotransmitters may explain the change in color vision.

From this study, patients taking either sodium valproate or carbamazepine did not record a change in visual field defect. This finding was similar to a study by Sorri et al. which demonstrated that epileptic patients taking sodium valproate do not develop visual field defects [18]. The mean deviation derived from the study was relatively similar to that of ours. In another study, Nousiainen et al. also demonstrated that patients taking carbamazepine did not show any visual field defects [20]. Although there are no visual field defects, patients from both groups showed a reduction of mean deviation from the general population. The changes can be attributed to side effect of medication or disease process itself.

All the epileptic patients recruited did not display any contrast sensitivity abnormalities. Thus statistical analysis was not performed. Other studies investigating the effect of AEDs on contrast sensitivity also failed to demonstrate any changes in contrast sensitivity [18,21].

There was no significant thinning of RNFL between the two groups, which is comparable to a study done by Lobefalo et al. [19]. Lobefalo et al. demonstrated that study subjects did not show significant RNFL thinning when comparing between three study groups (control, sodium valproate and carbamazepine). However, RNFL thinning has been documented in patients suffering from epilepsy. Factors which may influence RNFL thinning include duration of epilepsy, drug resistance and intellectual disability [22,23].

## Conclusions

There was no statistical significant difference in visual function between epileptic patients on sodium valproate and those on carbamazepine. However, changes of color vision and mean deviation recorded from the study population could indicate potential retina toxicity. Choice of anti-epileptic medication should be based on clinical judgment.
